# Epidemic Erysipelas, as It Occurred in Logansport (Indiana) and Its Vicinity, in 1845

**Published:** 1846-04

**Authors:** G. N. Fitch

**Affiliations:** Professor of Institutes and Practice of Medicine, in “Rush Medical College,” Chicago


ILLINOIS AND INDIANA

MEDICAL AND SURGICAL JOURNAL.

No. 1. NEW SERIES.—APRIL, 1846. Vol. I.

### PART I.—ORIGINAL COMMUNICATIONS.

### ARTICLE I.

*Epidemic Erysipelas*, as it occurred in Logansport (Indiana,) and its vicinity, in 1845. By G. N. Fitch, M.D., Professor of Institutes and Practice of Medicine, in "Rush Medical College," Chicago.

Considerable uniformity was observed in the manner of invasion. After a soreness of some superior portion of the neck, (internal or external,) of variable severity and duration, a rigor, in some cases slight, in others well marked, ushered in a more violent attack. The first local affection (of neck) was, in a majority of cases, internal; occupying the pharynx more particularly, but involving one or both tonsils, soft palate, and base of the tongue. There were, in these cases, more or less tumefaction of parts, increased vascularity, and redness of lining membranes, with difficulty and pain of swallowing; so great in some as to amount to almost total prohibition, the fluids regurgitating through the mouth or nose. In other cases there was manifest but little, if any, soreness of internal fauces; but tenderness, pain, and enlargement of cervical absorbents, sub-maxillary, and parotid gland of one side, or of sterno-mastoid muscle of one side in its upper third. In some few cases the rigor was the first evidence of disease; the local inflammation or pain not being developed until during it, or during the subsequent febrile excitement. The pulse, after the febrile reaction, became well established and rapid, 90 to 130, generally small, resisting and hard; variable, in those of same age and other apparent equal physical circumstances, in its rapidity and fullness; but in every instance, more rapid and smaller, and I may add, more corded than in our ordinary miasmatic fevers. Surface hot, though, in some, bathed with perspiration in a few hours after the disappearance of rigor; perspiration occurring thus early, not operating, however, as a relief to the patient, nor lessening the intensity of fever or heat of the skin. If the pharyngeal inflammation was severe, the lower extremities evinced a tendency to coldness. Tongue yellowish-white or brown. Nausea and vomiting attended the commencement of many cases. Thirst not very considerable, except in those violent cases in which deglutition was much impeded. Such, as I have described, was the manner of attack and symptoms, in by far the greater proportion. *(See* (f) *of tabular statement annexed.)* These symptoms constituted the entire disease in many; it yielding to treatment without involving any other part, and without the supervention of any other symptoms or changes, except such as indicated the gradual restoration of health. A portion, by no means small compared with the aggregate, were not, however, so fortunate, and the disease had a still further, though varied course to run. The inflammation occupying the pharynx and contiguous parts, would travel through the posterior nares, make its appearance externally, in the form of decided *erysipelas*, upon one *ala nasae*, or on the septum or tip of the nose, then spreading with varied rapidity over the face, forehead, scalp, and creeping down the neck to the superior portion of the trunk, and one or both upper extremities. Or it would reach the surface by the ear along the eustachian tube, and thence spread in the same manner over more or less extent. (h) Its appearance upon the external nose was preceded by a sense of fullness and heaviness of the frontal region, with obstruction of the nasal fossæ, and in some instances, discharge of bloody sanies. So likewise, there occurred, previous to its exhibition upon the external ear, deafness and pain in one or both ears, with, perhaps, the same sanious discharge. These symptoms, pain, fullness, and discharge from the parts, were not, in every instance, followed by external erysipelas. In some few cases the disease would be first visible, externally, at an angle of the mouth. Where the primary attack was in the cervical lymphatics, or parotid gland, or sterno-mastoid, the erysipelas of integuments usually commenced immediately over or near the part first affected. When it appeared externally, the inflammation was marked by vivid redness, considerable tumefaction, tenderness to touch, and hardness. Vesication was not constantly present; it was usually found in those cases where the tumefaction was greatest. When the palpabræ were reached in the progress of the inflammation, the swelling was so great as to close the eyes; the conjunctiva was rarely, if ever, involved.

Soreness of the neck was, in other instances, followed by erysipelas of integuments of superior, (i) or inferior (k) extremities, commencing usually on a finger, or toe; or by enlargement and pain of axillary glands, with, or without erysipelas of integuments in vicinity (l). In any situation where it existed, as the inflammation of the integuments spread, the tenderness to touch and hardness of parts previously traversed, gradually gave way to an uneasy tingling or itching sensation, and became œdematous (pitting) from effusion into cellular membranes beneath, and in the interstices of muscles. There was, at the same time, a diminution of redness, and desquamation commenced. In unfavorable cases, although desquamation in some degree, might commence, the original hardness and tenderness remained; the redness became livid—pulse increased in frequency, losing its hardness, and becoming thread-like, almost imperceptible; surface cool, and cold extremities, muttering delirium, or incoherency, or coma preceded a fatal result.

Acute Laryngitis supervened upon the primary attack in some, (m) constituting a formidable and distressing complication. The respiration, particularly inspiration, was long, laborious, croupy, with livid lips and face, starting eye-balls, and intense feeling of immediate suffocation, which too often quickly followed. Secondary inflammation of other organs or tissues, likewise followed the primary attack of neck or fauces, after an indeterminate period. Thus the bronchiæ (p) or pleura, (n) became the seat of inflammatory action. In females who had menstruated, peritonitis was of not uncommon occurrence, (s) independent of any condition of pregnancy. The dermoid tissue of vulva and contiguous parts were sometimes (t) seized with erysipelas. The pulse was varied little if any, by secondary inflammation of any organ or membrane; retaining its characteristic rapidity, smallness and hardness. In

*every instance* of the supervention of secondary symptoms, whether erysipelas of integuments, or inflammation of other parts, the original inflammation of fauces or neck, with its accompaniments, pain and tumefaction gradually disappeared. The subsidence of the first local symptoms never preceded, or was simultaneous with the secondary attack, so as to admit the supposition of metastasis. In some few cases the primary attack was not of the fauces or neck, but of the integuments of an extremity. Here, however, the symptom which can, I think, with propriety, be looked upon as characteristic of the disease, (soreness of the neck or fauces) was found present at some period of their progress.

Some attacks were, so far as could be ascertained, attributable solely to epidemic influence; the patients not having had any intercourse direct, or otherwise, previously, with any of the diseased. I do not entertain a doubt, however, of its contagious nature. By far the greater number of severe cases were traceable directly to this origin, and in turn communicated the disease to others; numerous examples, in proof of this, could be adduced. I shall be compelled, in an article condensed, as this is designed, to content myself with a few, well marked, —though not more unequivocal than many others. Two gentlemen, one resident twenty, and the other five miles distant, visited the house of a friend, four of whose family were confined with the disease in some stage. There had been no erysipelas in their respective neighborhoods;—none indeed nearer than at this point. They remained twelve or sixteen hours. In three days after their return both were seized with the disease. The attack of the one residing nearest proved mild, and it was not propagated to any extent from him. The other died; previously communicating it to almost every member of his father's large family;—one other of whom died also,—and from whom it spread over a considerable region, embracing a town of a thousand inhabitants.

A venerable lady 60 years old, residing distant ten miles in a sparsely settled vicinity, came into town to attend a sick married daughter, whose children had the erysipelas. She went home with it, or was seized immediately after her return. She had two other daughters, and three sons, married and living within one to three miles of her, and of each other;— yet so great was the terror of the disease from exaggerated reports of its fatality in town, that none evinced a willingness to become her attendant. Her aged husband was therefore nearly her sole one, until he was prostrate with the same. The elder daughter then became nurse for both: she was seized and died. Her husband, who had been at her bedside during her illness, was next attacked. The fear of the disease was naturally increased by these rapidly consecutive cases; and it was with great difficulty any could be found sufficiently courageous to perform the last offices for the dead, or administer to the wants of the sick. A sister-in-law of the deceased heroically volunteered her services; was soon taken and communicated it to her husband.

A nurse in attendance upon a family sick with it, was attacked went to her mother's, five miles distant, and spread it through her family.

The mother of a large family, four miles from town, washed bed-clothes and wearing apparel belonging to the family of a relative in town, among whom, then and previously, the disease was. She had a slight wound—a mere scratch, on the end of one finger, which had escaped her memory:—erysipelas attacked it, and spread over the arm, face, and scalp. During her illness, her family (five) were taken. The case of this mother is one of those, briefly alluded to before, in whom the primary attack was not of the neck or fauces; one of those parts, however, becoming affected in its farther progress. In all this (g) class of cases the disease was clearly contracted by inoculation. It is unnecessary to multiply cases in proof of its contagiousness. A sufficient number have been already mentioned to show the nature of the testimony upon which the entire absence of all "doubt" upon the subject, heretofore expressed, was based. I had seen the disease before; but not under circumstances to satisfy me of its contagiousness. When it first appeared here, therefore, my prejudices were strongly in favor of the negative side of the question. They were abandoned, only, after reiterated proof, afforded by observation, of their erroneousness. Some predisposition is, unquestionably, necessary for its contraction. Of those exposed to the contagion, only about one in two, or two and a half, appeared to be susceptible of its influence. In what the predisposition consists, we are, and probably, ever will remain, ignorant. Of the cases in the annexed statement, occurring in my practice, only a small number were under 12 years (a) of age. It must not hence be inferred, that childhood, and infancy operated as an immunity against the disease—only that the greater number had it very mild;—so mild, that no notes were taken of their cases. Of those above that age, more than half were females. (r) An obvious reason is presented for this, without supposing any peculiar predisposition in the sex, in the fact of their services being more frequently called into requisition in the capacity of nurses. How far having had it may protect against several attacks, I am not prepared, fully, to assert. From the result of very many second exposures, under apparently favorable circumstances for its contraction, my opinion is *strongly* biased in favor of one attack granting future exemption.

The sheet anchor, in treating it, is bleeding. This remedial agent was first resorted to cautiously, and with close attention to its effects. As the cases multiplied, thereby enlarging the field for observation and comparison of different modes of treatment, from its relatively much greater success, it was used boldly and freely. No remedy was as decidedly successful in so great number of cases,—no other remedy so frequently checked the disease—strangled it in its incipiency, or at least so modified its symptoms, as to render its subsequent course comparatively mild, when had recourse to at the proper time, and to such extent as the individual case demanded. The time for the lancet, was as soon as reaction, subsequent to the rigor, was fairly established; and the sooner thereafter, *ceteris paribus*, the greater, and more permanent, the curative impression. True, it was often required at uncertain subsequent periods, where it had, as well as where it had not been adopted early, in consequence of the supervention of secondary inflammation of some organ or tissue. Where thus indicated, the quantity was governed, as usual, by importance of the part seized, severity of symptoms, and relief obtained. After diminution of hardness of pulse, and increase of its frequency, with coolness of surface, and coldness of extremities, even when accompanied with delirium, or approach to coma, it became a doubtful remedy, although it may not have been previously used. Local bleedings were the safe depletory means here, if any were admissible. The benefit derivable from bleeding, was proportioned in an increase ratio, to the length of time that had elapsed since developement of the disease; and, except in those cases where demanded by secondary inflammation of vital tissues, it became doubtful, in a ratio directly proportioned to same lapse of time. I have bled beneficially as late as the fourteenth day; and have seen injury result as early as the fifth; and other cases where it was clearly inadmissible on the third. It will be seen that, of the cases between 12 and 21 (x) not bled, all recovered. They were mild throughout their progress, and in part, not seen until the period at which bleeding would be expected to beneficially break into the diseased train of action, had passed. So with those of greater age, not bled, or where original debility of habit, or advanced life forbade the remedy. Next to bleeding in importance, in the commencement, I rank emetics. Neither these nor bleeding should be resorted to in all cases, by routine or empirically, without, that is, the presence of symptoms which would afford satisfactory reasons for their use. Emetics were found particularly applicable to those cases accompanied with nausea, with or without vomiting, and with a yellowish-white tongue. Their repetition the second or third time, was demanded when there was difficult deglutition from viscid mucus in the inflamed pharynx; likewise when the larynx became the seat of inflammation. Under no other circumstances was their administration in the advanced stages followed by any compensating mitigation of symptoms. Cathartics were necessary throughout its entire course, but violent purging to be deprecated. I preferred, as a first cathartic, sub-chloride mercury, grs. 10 to 20, followed, in four to six hours, by ol. ricini, or sulph. magnesiæ. The repetition of sub-chloride was governed by appearance of the tongue and discharges. Care, however, should be taken, when the fauces were the seat of the local disease not to induce its constitutional effects. Ptyalism was productive of no good, except where there was existent secondary inflammation of peritoneum, bronchiæ, or lungs. Then its administration in alterative doses was decidedly indicated; these inflammations, particularly the first, yielding only to the combined influence of the lancet and mercurials, with such *adjuvants* as were called for by the individual case, or are most in vogue in acute diseases of these parts.

The mercurial, in peritonitis more especially, was usually combined with an opiate, where the lancet had been premised.

In the affection purely of the fauces, alterative mercurials were mischievous, and of doubtful efficacy in any other than the inflammatory complications above mentioned. When the inflammation was of the pharynx and fauces, counter irritants were applied to the neck—in milder cases, stimulating liniment; in the more severe, blisters. Various collutories were tried. All, however after a fair trial, and experience of their inefficiency, were discontinued, except dilute tinct. iodine.

*Tinct.* Iodine, ʒj

*Proof Spirit*, ʒvi

With this the patient was directed to gargle three to five times daily. In the urgent cases, attended with violent pyrexia, great pain, and difficult deglutition, the fauces were scarified, and bleeding encouraged, by gargles of warm water. The best means of discussing enlargement of the lymphatics and parotids and reducing the accompanying inflammation, was strong tinct. iodine, applied every four hours, by means of gentle friction, or of a piece of flannel moistened with it, and worn upon the neck as long as it could be borne, and renewed after the effect had partially subsided. In erysipelas of integuments resort was had to every plan of treatment offering a rational expectation of checking its progress and moderating symptoms. I speak more particularly of local,—the general treatment was that already described, modified by duration and severity of fever. Sol. nit. argent., tinct. iodine, oint. iodine, mercurial oint. and numerous other topical applications were made to inflamed surface in the first stage of the inflammation, that is, before effusion, the part being yet hard and painful. Certainly no good effect resulted, in any case, and in some the reverse. It was judged that emollient cataplasms would be more successful. They were tried, and found even more objectionable than the first named local means. Their inner surface dried, and became glued to the inflamed cuticle. When an effort was made to take them off for renewal, or any other purpose, dry crusts were found tenaciously adherent, which the attendants would either tear off with force and increase the inflammation and pain thereby; or leave to act as a constant local irritant upon an inflamed surface, and produce, what was actually the result in some cases, superficial sloughing. Mischief growing out of applications of this kind was still more apparent where there were vesications. The vesicles breaking, their contents would form, with the cataplasm, hard crusts which could not be removed without the application of considerable force. The only instances of gangrene of integuments to any extent, within my knowledge, were clearly attributable to the neglected presence of these crusts consequent upon the use of cataplasms as a domestic remedy, or in the hands of empirics. The only topical remedy, the success of which warranted its use in every case, in the phlegmonous stage, was a liniment of ol. lini. and aqua calcis, equal parts. This, when applied with a soft cloth or feathered end of a quill, allayed the burning, and communicated a grateful cooling sensation, not obtained by other means. To check the progress of the inflammation, nit. argent, in substance was used, by applying it to the contiguous sound skin, in such manner as to surround the diseased part, at least in the direction of its advance. In no instance did it fulfil the indication desired, in even a small degree; although applied freely, and frequently. The disease, in some instances, it is true, did not spread far beyond the cauterized line; but in such it was obvious, from its appearance, and the concomitant symptoms, that the inflammatory extension was nearly exhausted, and about reaching a spontaneous limitation.

I saw no reason for believing that the inflammation of integuments, while recent, and in full vigor, was checked in its onward advance, for a limited time, much less stopped, by this caustic. Blisters were applied in the manner usually directed, viz: by surrounding the diseased surface with a *cordon* of various strips, spread with the vesicating plaster, leaving a part only of each strip in contact with the sound skin. The effort to thus impede its march was soon ascertained to be nugatory. Before vesication, or indeed any considerable irritation, of the surface ensued, erysipelas was found occupying the integuments beyond the limits thus vainly attempted to assign to it. I, therefore, applied the strips sufficiently in advance to procure full vesication before the disease reached the surface covered by them,—say one half to one inch. In this manner, a complete stop was put to its farther progress; or, if it extended beyond, it was less violent; wanting the vivid redness, and tumefaction which characterized it within the blistered circumference. In the œdematous stage (after pitting) it became important to procure the absorption, or discharge of effused serum. For hastening the first process nothing equalled the topical application of tinct. iodine dilute, *(tinct. iodine* ʒ ij, *proof spirit* ʒ ij *to* ʒ jv*).* When its use every four hours, for 24 to 48 hours, failed to procure perceptible reduction of tumefaction; or when the latter was very considerable, it was not exclusively relied upon. Under such circumstances free incisions were made; or, the situation of the part forbidding that, punctures with a lancet. The use of these means early in the œdematous stage, prevented suppuration. When they were neglected, the effused serum, either from tension of its quantity, or its irritating quality, produced suppurative action,—broke down the cellular membrane—became extensively diffused in the interstices of muscles, and at last made its way externally through an ulcerated or excised opening, mingled with pus, and shreds of sphacelated cellular membrane. I am unwilling to admit the possibility of the occurrence of gangrene as a termination, unless from neglect, in the patient, of early seeking proper medical aid; or criminal mismanagement upon the part of the physician in not checking by proper treatment, the first violence of local inflammation, perhaps aggravating it by his topical applications; or in using no precautions against the effects reasonably expected to result from extensive extravasation of serum. Suppuration occasionally followed a glandular inflammation in the axilla, groin or mammæ, involving cellular membrane to some extent, but unaccompanied with erysipelas of superincumbent integuments. In such cases the secretion of pus was not preceded by any œdematous stage, and a circumscribed abscess was formed. If an extremity (hand or foot) was the seat of erysipelatous inflammation, permanent flexion, or extension of more or less of the digital tendons was a usual result; and this equally when the absorption of the serous extravasation was aided only by iodine, and when its discharge was promoted by punctures. Such contraction and partial rigidity were not, as has been supposed by many, caused by a breaking down of cellular membranes surrounding, and consequent agglutination of all parts, tendons, sheath, periostium, muscles and integuments. That it was not, was manifest from the part, (finger) enjoying some degree of mobility, during which it was evident the tendon, though shortened, moved within its theca, unconnected with skin, or bone. That it was not, was proved, moreover, by the result of several subcutaneous divisions of the affected tendons, after which the finger soon resumed very nearly its natural position. It is proper to remark that during the phlegmonous stage, the blisters were dressed with the same liniment, (ol. lini and aqua calcis) applied to the diseased surface; after œdema, while iodine was applied to the latter, the former were covered with mild blue ointment.

It remains only to notice that formidable variety of this disease,—Puerperal Peritonitis. It is unnecessary at this day to adduce any argument for establishing the identity of this disease, when epidemic, with erysipelas. Every intelligent physician admits it; and any attempt to so elucidate the matter as to convince the benighted understanding of him who does not, would be "casting pearls before swine." Another matter, however, connected with it is not so well understood, viz: its treatment. At least, if no more successful plan of treatment is ever to be adopted than the one now usually pursued, but a gloomy prospect is presented to those liable to its attacks. Its fatality probably exceeds that of any other acutely inflammatory disease. In Caledonia, Vt., of 30 cases, but one recovered. Twenty died in Bath, N. H., a town of only 1600 inhabitants, and here we infer that the 20 comprised all attacked, although the number of the latter is not stated. (*Vide* Amer. Jour., Jan., '44.) This, or very similar fatality, has attended it everywhere. It will be perceived that of the cases in the annexed table, nearly half died; and this notwithstanding, from the known prevalence of erysipelas, the peritonitis was expected, carefully watched for, and promptly met. Yet the most decisive treatment could not prevent the death of a large proportion. No treatment, hitherto practiced, has been attended with such success, as to encourage us much in its persistance; but of all *known* remedies, in the onset, none, in my estimation, can compare with bleeding. I say "*in the onset*," for if that golden moment is permitted to pass, the aid of the lancet had better not be invoked. Indeed, with my late experience, if I had not seen the patient, or had neglected to bleed in the *first few hours* after the rigor, in the more acute cases, I would dismiss the most remote idea of doing it at all. It will only hasten her tomb-bound journey. The severity of attack and danger are proportioned to the shortness of the period that elapses after accouchment before the rigor. If it makes its appearance in five hours, the onslaught is so violent, that only an imperfect reaction for a short time may follow; the symptoms marking the rapid approach of the last stage—collapse. In most cases a longer time—two or three days—in some cases even more, may supervene before attack. Then a longer period is presented during which bleeding may be required, although then the earlier a vein is opened, the greater the probability of recovery. I followed bleeding with cal. and opium, (gr. 1 to 2 of latter, and gr. 10 to 20 of former,) every four hours. After fourth or fifth dose, oil, if the bowels were not previously moved. Usually, it is true, a bland tepid enema was administered much earlier. The application of mercurial ointment with turpentine to the abdominal parieties, was directed all the time. It will be perceived that every one recovering was ptyalised. Cal. and opium, however, unless preceded by bleeding, did not control the disease, though given as above, or even more freely and frequently for two days or more. It appeared impossible to induce ptyalism without premising bleeding. The system had first to be *reduced* to a mercurial standard. Finding evidence of the constitutional effects of the mercurial an invariable precursor of an amelioration of all urgent symptoms, the disease appearing to give way before it, I was led to hope, that the administration of the same remedy in alterative doses, pushed so far as to give upon the gums mild, but unequivocal manifestations of its effect before accouchment, if kept up gently until that event, might protect against these peritoneal and uterine attacks. Accordingly, mass hydrarg. in grs. 2 to grs. 3 in pills, twice daily, conjoined in some instances with ung. hydrarg. ℨi, applied to abdomen every evening with friction, was recommended to several, at periods varying from two to four weeks previous to the expected full term of utero-gestation. In four cases in which the mercurial effect was manifest at the time of delivery, no symptom of peritonitis presented itself. One had the disease quite mildly. The pill was continued until the third or fourth day after accouchment. These few cases (five being all in which this plan was adopted,) do not afford sufficient ground for confident reliance upon the prophylactic power of the remedy. They create a supposition—encourage a *hope*—that it possesses such power; and fortunate, truly, for mothers, will it be if it does. Other and more extended trials will, I trust, be made by the profession as opportunities present, which will soon be decisive of the matter. In the four cases in the following table, laboring under erysipelas at the time of accouchment, two had it in the integuments, two in the fauces. In all, it was immediately checked upon supervention of peritonitis, requiring no further treatment and gradually disappearing. In no instance did a patient, who had erysipelas at any period during pregnancy, and recovered entirely before confinement, have peritonitis after that event.

The foregoing description of "epidemic erysipelas," is drawn entirely from personal observation; and the treatment such as that same observation and experience taught, in my estimation, to be requisite. For any opinions expressed I must hold myself solely responsible, as I have endeavored to be governed by the dictates of my own judgement, unbiased by any speculative notions of any theoretical or alleged therapeutical deductions of any practical writer.

SUMMARY.

Entire number of cases of which I took notes, in the counties of Cass, Fulton and Carroll. - - 213

(a) Between 6 months and 12 years of age, - 19
" 12 and 21 " " - 41
" 21 " 31 " " - 52
" 31 " 40 " " - 58
40 and over, - 43

(f) The primary attack was in fauces, tonsils, sterno-mastoid muscle or cervical glands in - - 182

(g) The disease clearly contracted by inoculation in some other part, (finger usually) in - - - 9

(h) Primary affection of fauces, &c. (f) was followed by erysipelas of face, scalp, and neck in - - 49

(i) By the same affection of integuments of arm and hand in - - - - - - 10

(k) By same in one or both lower extremities in 6

(l) By inflammation of axillary glands "with or without erysipelas of integuments in vicinity," in - 7

(m) By Laryngites in (6 males, 2 females, fatal in 6) 8

(n) By pleuntis in - - - - 2

(o) " meningitis in - - - - - 1

(p) By bronchitis alone, or complicated with pneumonia, in - - - - - - - 11

(q) By "black tongue" in - - - - 2

(r) Of the entire number above the age of 12, there was of females - - - - - - 102

(s) Of these the primary attack (f) was followed by peritonitis, independent of any condition of pregnancy, but in those who menstruated, in - - - - 15

(t) By secondary erysipelas of vulva and contagious parts, in - - - - - - - 5

By same of mamæ, in - - - 1

Of those under 12 years of age there was bled, (a bad case, but recovered.) - - - - - 1

Under that age (12) there died not bled. - 2

| | | |
|---|---|---|
| Between the age of 12 and 21 there were bled on 1st day, | | 17 |
| | of whom died | 0 |
| " " " " " | were bled on 2d " | 12 |
| | of whom died | 0 |
| " " " " | were bled on 3d day and after | 4 |
| | of whom died | 1 |

Of the remaining 8 not bled, none died.

| | | |
|---|---|---|
| Between 21 and 31 there were bled on | 1st day | 21 |
| | of whom died | 0 |
| " " " " " | 2d day | 15 |
| | of whom died | 1 |
| " " " " " | 3d day and after | 9 |
| | of whom died | 2 |

Of remaining 7 not bled, there died - - 2

| | | |
|---|---|---|
| Between 31 and 40 there were bled on 1st day | | 19 |
| | of whom died | 0 |
| " " " " " | 2d day | 15 |
| | of whom died | 2 |
| " " " " " | 3d day and after | 13 |
| | of whom died | 2 |

Of the remaining 11 not bled, there died - - 2

Over 40 there were bled on 1st day 11, of whom died 1

" " " " 2d day 14, " " 2

Of the remaining 18 not bled, there died - - 9

### Puerperal Peritonitis.

Cases, - - - - - - - 20

| | | | |
|---|---|---|---|
| Of these, there had erysipelas at time of accouchment | 4, | of whom died | 2 |
| Disease (peritonitis) forming before accouchment | | | 1 |
| Attacked on 1st day after— | 2, | of whom died | 2 |
| " 2d " " | 6, | " " | 3 |
| " 3d " " | 6, | " " | 2 |
| " 7th " " | 1, | | — |
| | | | 9 |
| Of all there were bled in from 3 to 12 hours after attack | 12, | of whom died | 3 |
| Bled on 2d day and subsequently | 6, | " " | 4 |
| Not bled - - - | 2, | " " | 2 |
| | | | — |
| | | | 9 |

Every one who recovered was mercuralized.

In two of these cases the infant was attacked with erysipelas of pudenda and neighboring parts on third day after birth, and died. Three were still born.

